# Horizontal and vertical optic disc rotation. The Beijing Eye Study

**DOI:** 10.1371/journal.pone.0175749

**Published:** 2017-05-08

**Authors:** Yuan Yuan Fan, Jost B. Jonas, Ya Xing Wang, Chang Xi Chen, Wen Bin Wei

**Affiliations:** 1 Beijing Tongren Eye Center, Beijing Tongren Hospital, Capital Medical University; Beijing Key Laboratory of Intraocular Tumor Diagnosis and Treatment, Beijing Ophthalmology and Visual Science Key Laboratory, Beijing, China; 2 Beijing Institute of Ophthalmology, Beijing Key Laboratory of Ophthalmology and Visual Sciences, Beijing Tongren Eye Center, Beijing Tongren Hospital, Capital Medical University; Beijing, China; 3 Department of Ophthalmology, Medical Faculty Mannheim of the Ruprecht-Karls-University, Mannheim, Germany; The University of Melbourne, AUSTRALIA

## Abstract

**Purpose:**

To measure the optic disc rotation around the vertical and horizontal disc axis and to evaluate associations with general and ocular parameters.

**Design:**

Population-based study

**Methods:**

In the Beijing Eye Study, 3468 participants (mean age:64.6±9.8 years; range:50–93 years) underwent an ophthalmological examination which included spectral-domain optical coherence tomography (OCT) with enhanced depth imaging. Using the OCT images, we determined the amount of the rotation of the optic disc (defined as Bruch´s membrane opening (BMO)) around the vertical axis and horizontal axis.

**Results:**

Optic disc rotation measurements were available for 3037 (87.6%) individuals. In multivariate analysis, larger optic disc rotation around the vertical axis (range:-4.90° to 41.0°) was associated (regression coefficient r:0.27) with high axial myopia (axial length ≥26.5 mm) (*P*<0.001;standardized regression coefficient beta beta:0.09), longer disc-fovea distance (*P* = 0.001;beta:0.09) and wider parapapillary beta/gamma zone (*P*<0.001;beta0.12). Larger optic disc rotation around the horizontal axis (range:-7.10° to 26.4°) was associated (r:0.32) with high axial myopia (*P* = 0.001;beta:0.08), larger optic disc-fovea angle (*P*<0.001;beta:0.13), thinner superior nasal retinal nerve fiber layer (RNFL) thickness (*P*<0.001;beta:-0.19) and thicker inferior nasal RNFL thickness (*P*<0.001;beta:0.17).

**Conclusions:**

Vertical optic disc rotation was associated with highly myopic axial elongation, increased disc-fovea distance and development or enlargement of parapapillary, Bruch´s membrane free, gamma zone, while macular Bruch´s membrane length is not affected. Horizontal optic disc rotation was associated with inferior dislocation of the fovea, in addition to a thinner superior nasal RNFL and thicker inferior nasal RNFL. The latter association may be taken into account in the interpretation of RNFL thickness profiles.

## Introduction

The morphology of the optic disc is characterized by the size and shape optic nerve head [1]. While prior to the clinical introduction of spectral-domain optical coherence tomography (OCT) the optic nerve head form was described according to its appearance upon two-dimensional ophthalmoscopy or on fundus photographs, OCT imaging now enables the three-dimensional assessment of the optic nerve head [2–7]. Previous studies have shown that using the OCT images allows the assessment of the rotation of the optic disc around the vertical axis, horizontal axis and sagittal axis [8]. The optic disc rotation around the vertical axis (usually with a backward movement of the temporal disc border and a forward movement of the nasal disc margin) results in a perspectively shortening of the horizontal disc diameter upon ophthalmoscopy. It leads to a change in the ophthalmoscopically perceived shape of the disc from a circular form to a vertically oval shape. Disc rotation around the horizontal axis (usually with a backward movement of the inferior disc border and a forward movement of the superior disc pole) is associated with a perspectively shortening of the vertical disc diameter so that a circular disc appears to be horizontally oval. Disc rotation around the sagittal axis is not associated with a ophthalmoscopic distortion. Since the OCT technology has become generally available, since particularities of the three-dimensional disc shape may be associated with other ocular and general parameters, and since population-based data on the three-dimensional optic nerve head shape have not been assessed yet, we conducted this study to examine the optic nerve head shape in a three-dimensional manner in a population-based manner and to correlate the three-dimensional disc shape with other, morphologic features of the optic nerve head.

## Methods

The Beijing Eye Study 2011 was performed in 2011 and had a population-based recruitment of study participants [9]. The protocol of the investigation was approved by the Medical Ethics Committee of the Beijing Tongren Hospital. Informed written consent was obtained from all participants. All study participants had to have an age of equal to or more than 50 years as the only inclusion criterion. There was no exclusion criterion. The number of 3468 individuals participating in the survey, out of 4403 individuals fulfilling the inclusion criterion, represented a participation rate of 78.8%. From the rural study region, 1633 (47.1%) subjects took part, while 1835 (52.9%) subjects came from the urban study region.

The study participants first underwent an interview conducted by trained research technicians. Using standardized question, this interview explored demographic parameters, the socioeconomic background, known major systemic diseases, and the cognitive function [10]. The blood concentrations of lipids, glucose and glycosylated hemoglobin HbA1c were determined in fasting blood samples. Additionally, we measured the blood pressure, body height and weight, and the waist and hip circumference.

The ophthalmological part of the survey consisted of automatic refractometry (Auto Refractometer AR-610, Nidek Co., Ltd, Tokyo, Japan) and visual acuity assessment (presenting visual acuity, uncorrected visual acuity, best corrected visual acuity), pneumo-tonometry for measurement of intraocular pressure (CT-60 computerized tonometer, Topcon Ltd., Japan), and photography of the anterior segment of the eye (Neitz CT-R camera (Neitz Instruments Co., Tokyo, Japan) and posterior segment of the eyes (Type CR6-45NM, Canon Inc. U.S.A.). On the photographs of the optic nerve head, we outlined the borders of the optic disc, optic cup and parapapillary gamma/beta zone [11–13]. The latter was defined as the parapapillary region with visible sclera [14,15]. The width of parapapillary beta / gamma zone was measured on a line connecting the disc center and the fovea. Beta / gamma zone was characterized by visible sclera and visible large choroidal vessels. If beta / gamma zone was present, it was located at the peripapillary ring of the optic nerve head. It usually was separated from the remaining retina by a parapapillary alpha zone. The latter showed an irregular hyperpigmentation and hypopigmentation. Applying the methods described by Littmann and Bennett, we corrected for the magnification of the optic disc images by the optic media of the eye [16,17]. We used the axial length as the basic principle ocular parameter for the calculations. Additionally, we carried out optical low-coherence reflectometry (Lenstar 900 Optical Biometer; Haag-Streit, Koeniz, Switzerland) for ocular biometry (for all right eyes) and spectral-domain optical coherence tomography (SD-OCT) (Spectralis; Heidelberg Engineering, Heidelberg, Germany) (for all eyes) with the enhanced depth imaging modality, for measurement of the retinal nerve fiber layer (RNFL) thickness. The distance from the optic disc center to the foveola, measured on a line connecting the disc center with the fovea, and the angle between the disc-fovea line and the horizontal line was determined on the fundus photographs [18]. A slit lamp examination carried out by an experienced ophthalmologist assessed lid abnormalities, Meibomian gland dysfunction, corneal disorders, and peripheral anterior chamber depth using van Herick’s method. Gonioscopy was routinely performed for all study participants. The degree of cataract was determined using the standardized lens photographs as described recently [19]. Diabetic retinopathy was diagnosed on the fundus photographs [20]. Fundus tessellation was defined as variation in the visibility of the large choroidal vessels and was differentiated into three grades [21].

**Fig 1:**
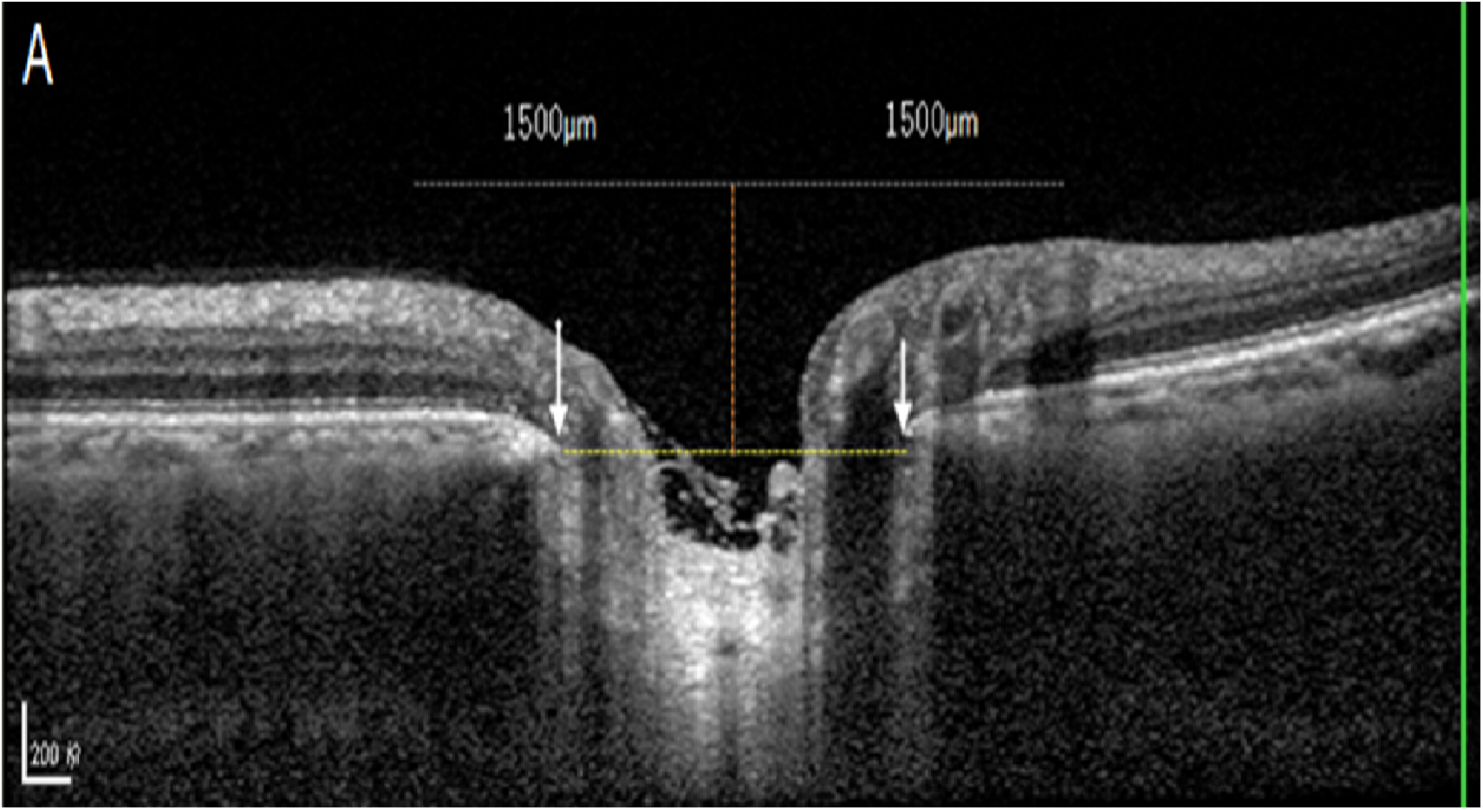
Optic coherence tomographic image of the optic nerve head: A perpendicular orange line was drawn through the middle of the yellow dash line connecting the inner edges of neural canal opening (delineated by white arrows).

**Fig 2:**
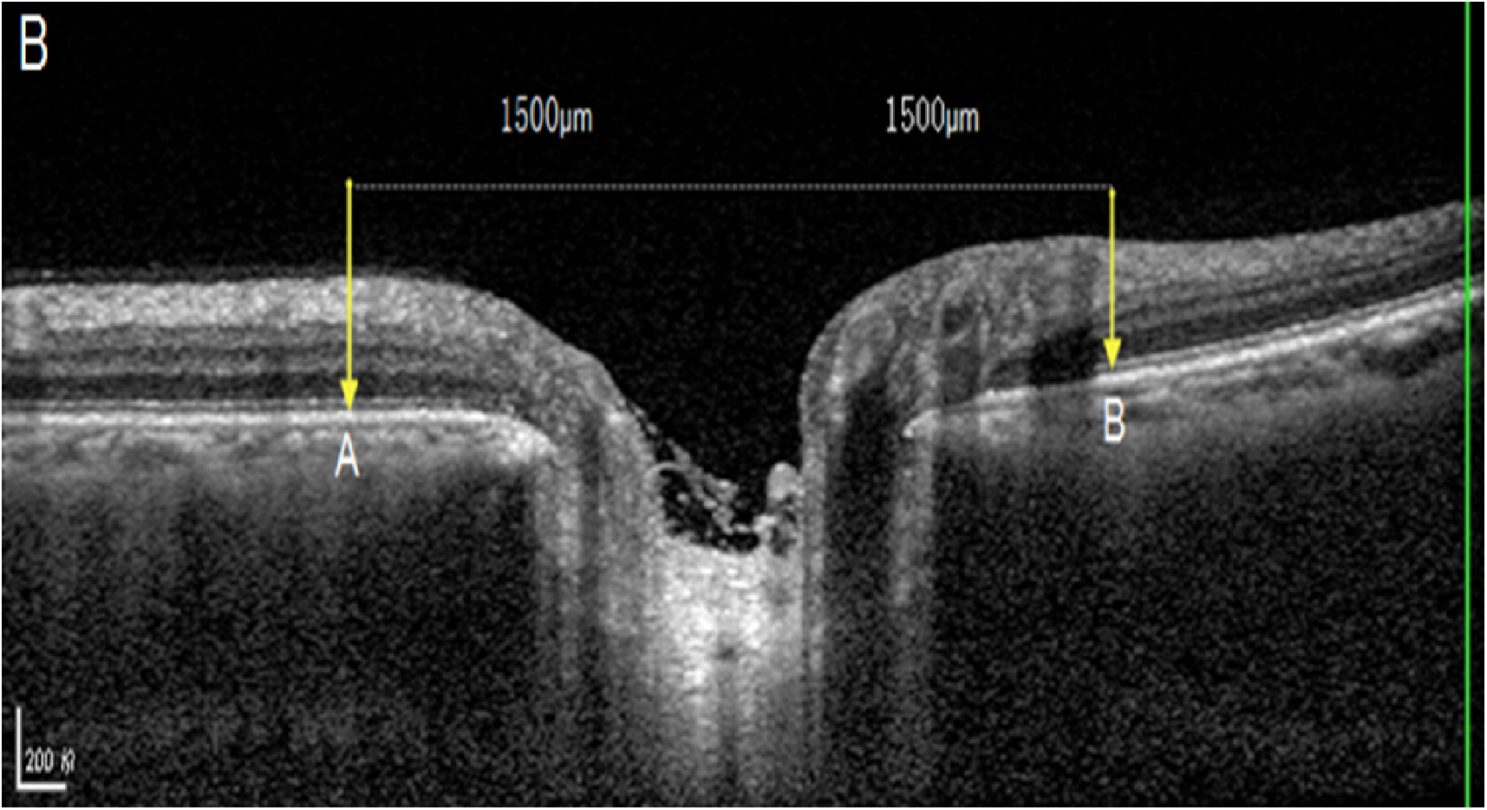
Optic coherence tomographic image of the optic nerve head: On each side of the orange line, a line was drawn (gray line) with a length of 1500 μm to each side. The two ends of this line (yellow vertical arrows) marked the two ends of the retinal pigment epithelium / Bruch´s membrane plane (between points A and B).

**Fig 3:**
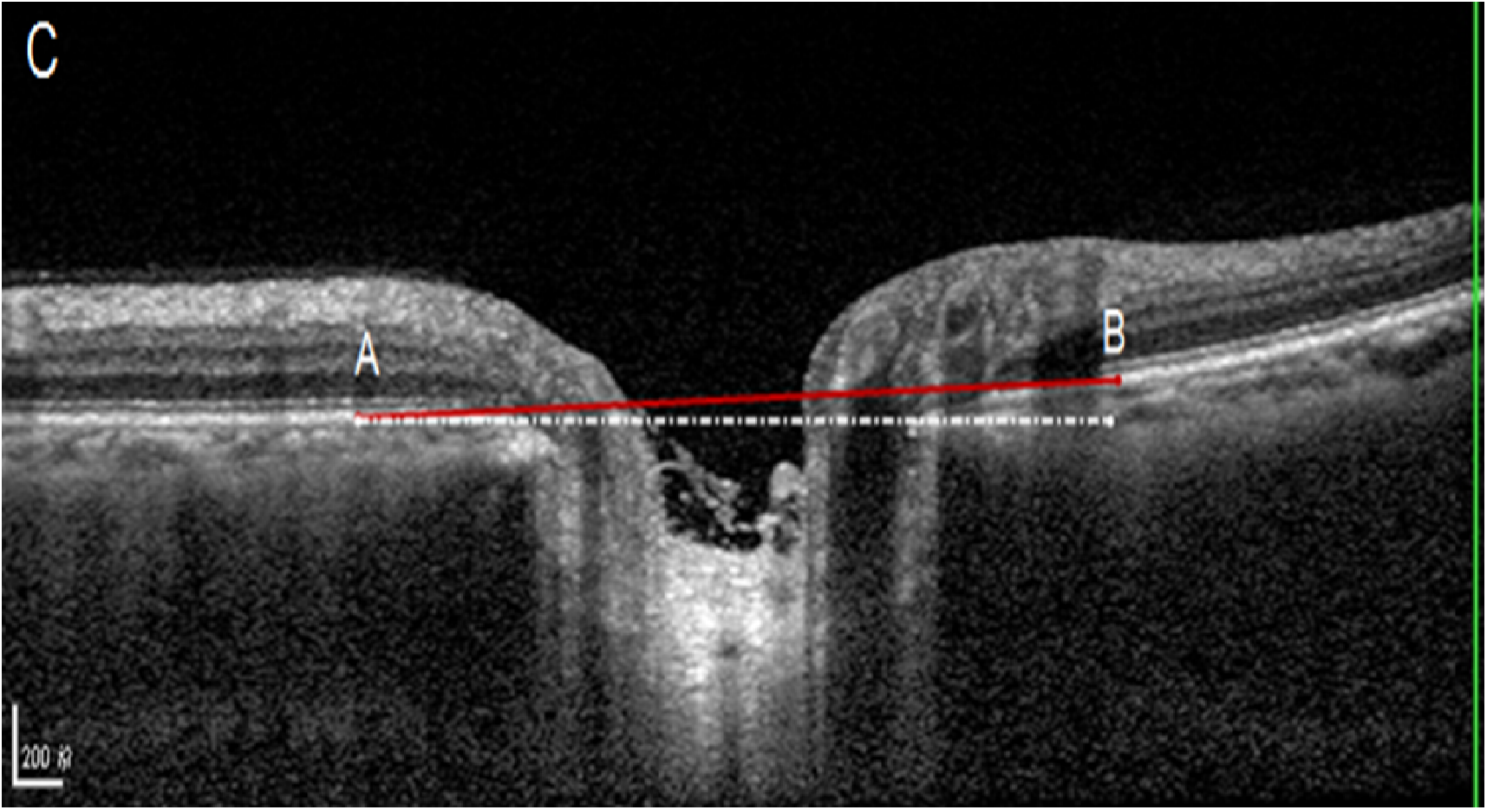
Optic coherence tomographic image of the optic nerve head: The Bruch´s membrane opening reference plane was defined as the red line between “A” and “B”. The angle between this line and the horizontal was defined as the angle of the optic disc rotation (as based on Bruch´s membrane opening plane).

Out of the 6 radial OCT B-scans which were obtained for the optic discs, we selected the horizontal scan and vertical scan running through the center of the optic disc for further analysis (Figs 1–3). The selected images were imported into ImageJ (1.47 National Institutes of Health, Bethesda, Maryland, USA; available at: “http://rsb.info.nih.gov//ij”). The inner borders of the optic disc neural canal opening were identified (Figs 1–3), and a line (“optic disc neural canal opening line“) connecting both borders was drawn. We measured the angle between this line and a line running horizontally to the OCT image and called this angle “optic disc neural canal opening angle”. A second line (“Bruch´s membrane line”) was drawn starting and ending at the level of Bruch´s membrane / retinal pigment epithelium at a distance of 1500 μm on each side of the middle point of the optic disc neural canal opening line. We measured the angle between this Bruch´s membrane line and a line running horizontally to the OCT image and called this angle “Bruch´s membrane opening angle” (Figs 1–3).

Optic disc rotation around the vertical axis (“vertical optic disc rotation”) was measured as the angle between Bruch´s membrane line and the OCT image line on OCT scans running horizontally through the optic disc. Optic disc rotation around the horizontal axis (“horizontal optic disc rotation”) was measured as the angle between Bruch´s membrane line and the OCT image line on OCT scans running vertically through the optic disc. The vertical disc rotation angle was positive if the nasal optic disc border was elevated and the temporal disc border was depressed. The horizontal disc rotation angle was positive if the superior optic disc border was elevated and the inferior disc border was depressed. Similar measurements were performed for the optic disc neural canal opening angle in the horizontal direction and in the vertical direction. We additionally calculated the difference between the Bruch´s membrane opening angle and the optic disc neural canal opening angle and called it “angle difference”. High axial myopia was defined by an axial length of ≥26.5 mm.

Using the enhanced depth imaging mode, we additionally obtained seven OCT sections (each comprising 100 averaged scans) in a 5° x 30° large rectangle centered onto the fovea. Using the scan running through the foveola we measured for a subset of eyes the length of macular Bruch´s membrane defined as the distance to the foveola to the end of Bruch´s membrane in direction of the optic nerve head. The width of parapapillary gamma zone was defined as the distance between the end of Bruch´s membrane and the optic disc border.

We used a statistical software package (SPSS for Windows, version 22.0, IBM-SPSS, Chicago, IL, USA) for the statistical analysis. Only the right eye of each study participant was assessed. We calculated the mean values (mean ± standard deviation) of the disc rotation angles as the main outcome parameters. We assessed associations between the disc rotation angles and other ocular or general parameters in a univariate analysis. Finally, we carried out a multivariate analysis with the disc rotation angles as the dependent variable. We calculated the standardized regression coefficient beta, the non-standardized regression coefficient B and the 95% confidence intervals (CI). All *P*-values were 2-sided and were taken as statistically significant, if they were <0.05.

## Results

Optic disc rotation measurements were available for 3037 (87.6%) individuals (1715 (56.5%) women) out of the total 3468 participants. The group of individuals with measurements of the optic disc rotation and the group of subjects not included into the present study was significantly (*P*<0.001) younger (64.0 ± 9.5 versus 69.0 ± 10.7 years), had a better best-corrected visual acuity (0.04 ± 0.15 logMAR versus 0.21 ± 0.38; *P*<0.001), were less myopic (-0.15 ± 1.94 diopters versus -0.89 ±3.28 diopters; *P*<0.001), had shorter axial length (23.2 ± 1.1 versus 23.5 ± 1.4 mm; *P* = 0.002), and did not vary significantly in gender (*P* = 0.68). Reasons why SD-OCT images for the measurement of the optic disc rotation were not available were cataract and other causes for an insufficient quality of the images for a reliable determination of the disc rotation. Any ocular disease, including disorders of the optic nerve or macula, was no reason to exclude any subject if the quality of OCT image was sufficient to be evaluated.

Mean vertical optic disc rotation (based on the Bruch´s membrane opening angle) was 1.80 ± 2.21° (median: 1.58°; range: -4.90° to 41.0°), and the mean horizontal optic disc rotation (based on the Bruch´s membrane opening angle) was 1.75 ± 2.89° (median: 1.51°; range: -7.10° to 26.4°). Both angles did not differ significantly from each other (*P* = 0.57).

In univariate analysis, the vertical optic disc rotation was associated (*P*<0.05) with the systemic parameters of younger age, female gender, and smaller waist circumference, and with the ocular parameters of longer axial length, axial length ≥26.5 mm, more myopic refractive error, shorter corneal curvature radius, smaller optic disc area, wider parapapillary beta / gamma zone (as both zones measured together), wider parapapillary gamma zone, longer disc-fovea distance, thinner RNFL thickness overall and in the nasal superior region, nasal region, nasal inferior region, thicker RNFL thickness in the temporal region, thinner subfoveal choroidal thickness, and lower degree of fundus tessellation overall, in the macular region and in the peripapillary region (Table 1). Vertical optic disc rotation was not significantly associated with the systemic parameters of region of habitation (*P* = 0.97), systolic (*P* = 0.78) and diastolic (*P* = 0.30) blood pressure, body mass index (*P* = 0.39), smoking package years (*P* = 0.71), cognitive function score (*P* = 0.07; beta: 0.03) and the blood concentration of high-density lipoproteins (*P* = 0.81), low-density lipoproteins (*P* = 0.07), cholesterol (*P* = 0.12) and glucose (*P* = 0.78), and the ocular parameters of central corneal thickness (*P* = 0.27), corneal diameter (*P* = 0.56), anterior chamber depth (*P* = 0.24), lens thickness (*P* = 0.73), size of parapapillary alpha zone (*P* = 0.52), disc-fovea angle (*P* = 0.58), macular Bruch´s membrane length (*P* = 0.08), cylindrical refractive error (*P* = 0.34), RNFL thickness in the superior temporal region (*P* = 0.09), superior region (*P* = 0.17), inferior region (*P* = 0.35) and inferior temporal region (*P* = 0.05), intraocular pressure (*P* = 0.49), best corrected visual acuity (*P* = 0.45), and prevalence of open-angle glaucoma (*P* = 0.41), angle-closure glaucoma (*P* = 0.82), retinal vein occlusions (*P* = 0.45), diabetic retinopathy (*P* = 0.30), and early (*P* = 0.18), intermediate (*P* = 0.49) and late (*P* = 0.93) age-related macular degeneration.

**Table 1:** Univariate analysis of associations between the vertical optic disc rotation (°) (as measured by the Bruch´s membrane opening angle to the horizontal) and systemic and ocular parameters in the Beijing Eye Study.

Parameter	*P-*Value	Standardized Coefficient Beta
Age (Years)	<0.001	-0.08
Men / Women	0.005	0.05
Waist Circumference (cm)	0.03	-0.04
Axial Length (mm)	<0.001	0.08
Refractive Error (Diopters)	<0.001	-0.14
Corneal Curvature Radius (mm)	0.04	-0.04
Optic Disc Area (mm^2^)	0.03	-0.05
Parapapillary Beta/Gamma Zone Width (mm)	<0.001	0.12
Parapapillary Gamma Zone Width (mm)	<0.001	0.20
Disc-Fovea Distance (mm)	0.04	0.04
Retinal Nerve Fiber Layer Thickness (μm), Overall	0.001	-0.06
Retinal Nerve Fiber Layer Thickness (μm), Nasal Superior	0.001	-0.06
Retinal Nerve Fiber Layer Thickness (μm), Nasal	0.003	-0.05
Retinal Nerve Fiber Layer Thickness (μm), Nasal Inferior	0.03	-0.04
Retinal Nerve Fiber Layer Thickness (μm), Temporal	0.02	0.04
Subfoveal Choroidal Thickness (μm)	<0.001	0.12
Fundus Tessellation Degree, Overall	<0.001	-0.09
Fundus Tessellation Degree, Macular Region	<0.001	-0.07
Fundus Tessellation Degree, Peripapillary Region	<0.001	-0.10

In the multivariate analysis with the vertical optic disc rotation as dependent variable, we first dropped the degree of fundus tessellation in the peripapillary region (variance inflation factor (VIF): 9.7) and in the macular region (VIF: 14.7), total RNFL thickness (VIF: 6.4), refractive error (VIF: 4.6), and gamma zone width (due to collinearity with beta/gamma zone width; VIF: 2.58). Due to lack of statistical significance, we then dropped step by step age (*P* = 0.94), axial length (*P* = 0.92), nasal inferior RNFL thickness (*P* = 0.87), temporal RNFL (*P* = 0.82), superior nasal RNFL (*P* = 0.64), nasal RNFL (*P* = 0.43), and corneal curvature radius (*P* = 0.06). In the remaining final model (regression coefficient r: 0.27), larger vertical optic disc rotation was associated with the systemic parameters of female gender (*P* = 0.002) and smaller waist circumference (*P* = 0.02), and with the ocular parameters of longer disc-fovea distance (*P* = 0.001), wider beta/gamma zone (*P*<0.001), smaller optic disc area (*P* = 0.005), axial length ≥26.5 mm (*P*<0.001), thicker subfoveal choroidal thickness (*P*<0.001), and lower degree of fundus tessellation (*P*<0.001) (Table 2). In a similar manner, if instead of the beta/gamma zone width the width of gamma zone as measured by OCT was included into the statistical analysis, larger vertical optic disc rotation was significantly associated with wider gamma zone (*P*<0.001; beta: 0.30; B: 0.005; 95%CI: 0.002, 0.007). If the length of macular Bruch´s membrane as measure don the OCT images was added to the model, it was not significantly (*P* = 0.85) associated with the vertical optic disc rotation.

**Table 2:** Univariate analysis of associations between the horizontal optic disc rotation (°) (as measured by the Bruch´s membrane opening angle to the vertical) and systemic and ocular parameters in the Beijing Eye Study.

Parameter	*P-*Value	Standardized Coefficient Beta
Age (Years)	<0.001	0.09
Rural / Urban Region of Habitation	<0.001	0.09
Body Mass Index (kg/m^2^)	0.02	-0.04
Axial Length (mm)	<0.001	0.15
Refractive Error (Diopters)	<0.001	-0.11
Cylindrical Refractive Error (Diopters)	<0.001	0.07
Corneal Curvature Radius (mm)	0.02	0.04
Parapapillary Alpha Zone (mm^2^)	0.001	0.07
Parapapillary Beta/Gamma Zone Width (mm)	<0.001	0.09
Disc-Fovea Distance (mm)	0.007	0.05
Disc-Fovea Angle (°)	<0.001	0.18
Macular Bruch´s Membrane Length (mm)	<0.001	-0.10
Retinal Nerve Fiber Layer Thickness (μm), Overall	0.002	-0.06
Retinal Nerve Fiber Layer Thickness (μm), Temporal Superior	<0.001	-0.10
Retinal Nerve Fiber Layer Thickness (μm), Superior	<0.001	-0.11
Retinal Nerve Fiber Layer Thickness (μm), Nasal Superior	<0.001	-0.18
Retinal Nerve Fiber Layer Thickness (μm), Nasal	<0.001	-0.18
Retinal Nerve Fiber Layer Thickness (μm), Nasal Inferior	0.04	-0.04
Subfoveal Choroidal Thickness (μm)	<0.001	-0.17
Fundus Tessellation Degree, Overall	<0.001	0.15
Fundus Tessellation Degree, Macular Region	<0.001	0.15
Fundus Tessellation Degree, Peripapillary Region	<0.001	0.14
Best Corrected Visual Acuity (logMAR)	0.02	0.04
Intermediate Age-Related Macular Degeneration, Prevalence	0.02	-0.04

In univariate analysis, the horizontal optic disc rotation was associated with the systemic parameters of older age, urban region of habitation, and lower body mass index, and with the ocular parameters of longer axial length, axial length ≥26.5 mm, more myopic refractive error, larger cylindrical refractive error, longer corneal curvature radius, larger parapapillary alpha zone, wider parapapillary beta / gamma zone (as both zones measured together), longer disc-fovea distance, wider disc-fovea angle, shorter macular Bruch´s membrane length, thinner RNFL thickness overall and in the temporal superior region, superior region, nasal superior region, nasal region, and nasal inferior region, thinner subfoveal choroidal thickness, higher degree of fundus tessellation overall, in the macular region and in the peripapillary region, lower best corrected visual acuity, and lower prevalence of intermediate age-related macular degeneration (Table 3). Horizontal optic disc rotation was not significantly associated with the systemic parameters of gender (*P* = 0.10), systolic (*P* = 0.42) and diastolic (*P* = 0.22) blood pressure, waist circumference (*P* = 0.17), smoking package years (*P* = 0.83), cognitive function score (*P* = 0.60) and the blood concentration of high-density lipoproteins (*P* = 0.14), low-density lipoproteins (*P* = 0.48), cholesterol (*P* = 0.36) and glucose (*P* = 0.61), and the ocular parameters of central corneal thickness (*P* = 0.18), corneal diameter (*P* = 0.98), anterior chamber depth (*P* = 0.08), lens thickness (*P* = 0.45), optic disc area (*P* = 0.64), parapapillary gamma zone (*P* = 0.26), RNFL thickness in the inferior region (*P* = 0.29) and temporal inferior region (*P* = 0.92), intraocular pressure (*P* = 0.14), and prevalence of open-angle glaucoma (*P* = 0.17), angle-closure glaucoma (*P* = 0.73), retinal vein occlusions (*P* = 0.68), diabetic retinopathy (*P* = 0.30), and early (*P* = 0.37) and late (*P* = 0.33) age-related macular degeneration.

**Table 3:** multivariate analysis of associations between the vertical optic disc rotation (°) (as measured by the Bruch´s membrane opening angle to the horizontal) and systemic and ocular parameters in the Beijing Eye Study.

	*P-*value	Standardized Coefficient Beta	Unstandardized Coefficient B	95% Confidence Interval for B	Variance Inflation Factor
Gender	0.002	0.07	0.29	0.10, 0.47	1.12
Waist Circumference (cm)	0.02	-0.05	-0.01	-0.02, -0.002	1.04
Axial Length ≥26.5 mm	<0.001	0.09	2.03	1.07, 3.00	1.09
Parapapillary Beta / Gamma Zone Area (mm^2^)	<0.001	0.12	0.31	0.19, 0.42	1.18
Optic Disc-Fovea Distance (mm)	0.001	0.09	0.50	0.22, 0.79	1.24
Optic Disc Area (mm^2^)	0.005	-0.06	-0.26	-0.44, -0.08	1.02
Fundus Tessellation Degree	<0.001	-0.10	-0.18	-0.28, -0.08	1.75
Subfoveal Choroidal Thickness (μm)	<0.001	0.14	0.003	0.002, 0.004	1.74

In the multivariate analysis with the horizontal optic disc rotation as dependent variable, we first dropped disc-fovea distance (VIF: 58.6), total RNFL thickness (VIF: 4.4), refractive error (VIF: 2.7), fundus tessellation degree in the macular region (VIF: 2.9) and in the peripapillary region (VIF: 7.3), and macular Bruch´s membrane length (VIF: 3.82; *P*: 0.87). Due to lack of statistical significance, we then dropped step by step body mass index (*P* = 0.85), age (*P* = 0.57), prevalence of intermediate age-related macular degeneration (*P* = 0.24), temporal superior RNFL thickness (*P* = 0.52), superior RNFL thickness (*P* = 0.16), axial length (*P* = 0.11), corneal curvature radius (*P* = 0.39), nasal RNFL thickness (*P* = 0.17), best corrected visual acuity (*P* = 0.20), parapapillary beta zone size (*P* = 0.14), and cylindrical refractive error (*P* = 0.22). In the remaining final model (regression coefficient r: 0.32), larger horizontal optic disc rotation was associated with the systemic parameters of rural region of habitation (*P* = 0.001) and with the ocular parameters of axial length ≥26.5 mm (*P* = 0.001), larger parapapillary alpha zone (*P* = 0.008), larger optic disc-fovea angle (*P*<0.001), thinner superior nasal RNFL thickness (*P*<0.001), thicker inferior nasal RNFL thickness (*P*<0.001), thinner subfoveal choroidal thickness (*P* = 0.02), and higher degree of fundus tessellation (*P* = 0.003) (Table 4).

**Table 4:** Multivariate analysis of associations between the horizontal optic disc rotation (°) (as measured by the Bruch´s membrane opening angle to the vertital) and systemic and ocular parameters in the Beijing Eye Study.

	*P-*value	Standardized Coefficient Beta	Unstandardized Coefficient B	95% Confidence Interval for B	Variance Inflation Factor
Rural / Urban Region of Habitation	0.001	0.07	0.40	0.16, 0.64	1.07
Axial Length ≥26.5 mm	0.001	0.08	2.21	0.95, 3.48	1.03
Parapapillary Alpha Zone Area (mm^2^)	0.008	0.06	0.30	0.08, 0.51	1.02
Optic Disc-Fovea Angle (°)	<0.001	0.13	0.10	0.07, 0.14	1.05
Retinal Nerve Fiber Layer Thickness Superior Nasal (μm)	<0.001	-0.19	-0.03	-0.03, -0.02	1.39
Retinal Nerve Fiber Layer Thickness Inferior Nasal (μm)	<0.001	0.17	0.02	0.01, 0.03	1.38
Fundus Tessellation Degree	0.003	0.08	0.20	0.07, 0.33	1.66
Subfoveal Choroidal Thickness (μm)	0.02	0.07	-0.002	-0.003, 0.000	1.71

The mean difference of the vertical rotation of Bruch´s membrane opening minus the vertical rotation of the optic disc neural canal opening (as measured on the horizontal OCT images (1.36 ± 2.01°; median: 1.16°; range: -10.0° to 36.25°) was significantly (*P*<0.001) larger than the mean difference of the horizontal rotation of Bruch´s membrane opening minus the vertical rotation of the optic disc neural canal opening (as measured on the vertical OCT image (-0.07 ± 1.27°; median: -0.05°; range: -16.0° to 17.94°). Correspondingly, the vertical rotation of Bruch´s membrane opening (1.80 ± 2.21°) was significantly (*P*P<0.001) larger than the vertical rotation of the optic disc neural canal opening (0.44 ± 0.90°). In univariate analysis, the difference in the rotation around the vertical axis was associated with younger age (*P*<0.001; beta: -0.09), female gender (*P* = 0.02; beta: 0.04), longer axial length (*P*<0.001; beta: 0.09), more myopic refractive error (*P*<0.001; beta: -0.14), thinner subfoveal choroidal thickness (*P*<0.001; beta: -0.11), larger parapapillary alpha zone (*P* = 0.002; beta: 0.13), larger parapapillary beta zone (*P*<0.001; beta: 0.13), shorter waist circumference (*P* = 0.007; beta: -0.05), smaller optic disc area (*P* = 0.02; beta: -0.05), lower degree of fundus tessellation (*P*<0.001; beta: -0.09), thinner RNFL overall (*P*<0.001; beta: -0.07), thicker RNFL temporal (*P* = 0.01; beta: 0.05), thinner RNFL nasal superior (*P*<0.001; beta: -0.08), nasal (*P* = 0.008; beta: -0.05), nasal inferior (*P*<0.001; beta: -0.06), inferior (*P* = 0.03; beta: -0.04), and temporal inferior (*P* = 0.009; beta: -0.05), longer parapapillary gamma zone (*P* = 0.02; beta: 0.14), longer disc-fovea distance (*P* = 0.001; beta: 0.07), and higher prevalence of high myopia (defined as axial length ≥26.5mm) (*P*<0.001; beta: 0.18).

In the multivariate analysis (r: 0.28), the angle difference in the rotation around the vertical axis remained to be significantly associated with longer disc-fovea distance (*P*<0.001), larger parapapillary beta zone (*P*<0.001), high axial myopia (*P*<0.001), female gender (*P* = 0.001), thicker subfoveal choroidal thickness (*P*<0.001), smaller optic disc size (*P* = 0.02), lower degree of fundus tessellation (*P*<0.001), thinner RNFL thickness inferior temporal (*P* = 0.02) (Table 5).

**Table 5:** Multivariate analysis of associations of the difference in the vertical rotation of Bruch´s membrane opening minus the vertical rotation of the optic disc neural canal opening and systemic and ocular parameters in the Beijing Eye Study.

	*P-*value	Standardized Coefficient Beta	Unstandardized Coefficient B	95% Confidence Interval for B	Variance Inflation Factor
Optic Disc-Fovea Distance (mm)	<0.001	0.10	0.54	0.28, 0.80	1.26
Parapapillary Beta / Gamma Zone Area (mm^2^)	<0.001	0.12	0.27	0.16, 0.37	1.17
Axial Length ≥26.5 mm	<0.001	0.10	1.94	1.07, 2.81	1.10
Gender	0.001	0.08	0.28	0.12, 0.45	1.12
Subfoveal Choroidal Thickness (μm)	<0.001	-0.15	-0.003	-0.002, -0.004	1.75
Inferior Temporal Retinal Nerve Fiber Layer Thickness (μm)	0.02	-0.05	-0.004	-0.008, -0.001	1.08
Fundus Tessellation Degree	<0.001	-0.10	-0.16	-0.25, -0.07	1.76
Optic Disc Area (mm^2^)	0.02	-0.05	-0.20	-0.36, -0.04	1.02

In univariate analysis, the difference in the rotation around the horizontal axis was associated with older age (*P*<0.001; beta: 0.10), male gender (*P*<0.001; beta: -0.07), longer axial length (*P*<0.001; beta: 0.13), more myopic refractive error (*P*<0.001; beta: -0.14), thinner subfoveal choroidal thickness (*P*<0.001; beta: -0.16), larger parapapillary alpha zone (*P* = 0.002; beta: 0.09), larger parapapillary beta zone (*P*<0.001; beta: 0.08), higher degree of fundus tessellation (*P*<0.001; beta: 0.20), longer corneal curvature radius (*P* = 0.01; beta: 0.05), deeper anterior chamber depth (*P*<0.001; beta: 0.07), thinner RNFL overall (*P*<0.001; beta: -0.07), thinner RNFL superior (*P* = 0.001; beta: -0.06), nasal superior (*P*<0.001; beta: -0.09), nasal (*P* = 0.04; beta: -0.04), nasal inferior (*P* = 0.01; beta: -0.05), inferior (*P* = 0.005; beta: -0.05), and temporal inferior (*P* = 0.001; beta: -0.06), longer disc-fovea distance (*P* = 0.009; beta: 0.05), shorter macular Bruch´s membrane length (*P* = 0.005; beta: -0.06), larger cylindrical refractive error (*P* = 0.03; beta: 0.04), and higher prevalence of high myopia (defined as axial length ≥26.5mm) (*P*<0.001; beta: 0.07).

In the multivariate analysis (r: 0.28), the angle difference in the rotation around the horizontal axis remained to be significantly associated with larger disc-fovea angle (*P* = 0.001), deeper anterior chamber depth (*P*<0.001), higher degree of fundus tessellation (*P*<0.001), and larger parapapillary alpha zone (*P* = 0.007) (Table 6).

**Table 6:** Multivariate analysis of associations of the difference in the horizontal rotation of Bruch´s membrane opening minus the horizontal rotation of the optic disc neural canal opening and systemic and ocular parameters in the Beijing Eye Study.

	*P-*value	Standardized Coefficient Beta	Unstandardized Coefficient B	95% Confidence Interval for B	Variance Inflation Factor
Optic Disc-Fovea Angle (°)	0.001	0.07	0.03	0.01, 0.04	1.00
Parapapillary Alpha Zone Area (mm^2^)	0.007	0.06	0.13	0.04, 0.23	1.01
Fundus Tessellation Degree	<0.001	0.18	0.19	0.15, 0.24	1.01
Anterior Chamber Depth (mm)	<0.001	0.08	0.22	0.10, 0.34	1.01

## Discussion

In our population-based study, the amount of the vertical optic disc rotation was associated with a longer disc-fovea distance, wider beta/gamma zone and presence of high axial myopia in a multivariate analysis. The horizontal optic disc rotation increased with larger optic disc-fovea angle, presence of high axial myopia, thinner superior nasal RNFL thickness and thicker inferior nasal RNFL thickness. The mean difference in the vertical rotation of Bruch´s membrane opening to the optic disc neural canal opening was significantly larger than the mean difference in the horizontal rotation of Bruch´s membrane opening to the optic disc neural canal opening. It indicated that the rotation around the vertical axis was more marked for Bruch´s membrane opening than it was for the optic nerve canal opening, while for the rotation around the horizontal disc axis, there was no marked difference between both planes. The mean difference in the vertical rotation of Bruch´s membrane opening to the optic disc neural canal opening was not correlated with disc-fovea distance or beta/gamma zone area.

These findings measured in our population-based investigation are in agreement with results obtained in previous hospital-based studies. The mean vertical optic disc rotation of 1.80 ± 2.21° as found in our study is similar to the median value of 3.5° as found in the study by Hosseini and colleagues or as in other investigations [7,22,23]. In a study by Dai and colleagues on selected myopic individuals with a mean axial length of 26.42 ± 2.60 mm, the mean optic disc rotation around the vertical axis was 14.4 ± 9.3° [8], the horizontal optic nerve head diameters were longer when determined on OCT images than as if measured on optic nerve head photographs, and this difference between the OCT-based determinations and the measurements on the disc photographs enlarged with longer axial length. The results of Dai´s study and our investigation suggest that vertically oval discs (as they appear upon ophthalmoscopy) are rotated around the vertical axis with the temporal disc side moving backwards. These observations confirm the findings made in other studies [18,22,23]. As in our study, the investigation by Hosseini and associates and by others revealed that the disc rotation angle was correlated with longer axial length [22,24,25].

Our study additionally reported that the vertical disc rotation was correlated with a wider beta / gamma zone and with a longer disc-fovea distance while it was not correlated with the macular Bruch´s membrane length. In a similar manner, a previous study revealed that a wider width of parapapillary gamma zone was correlated with longer axial length, more marked vertical optic disc rotation, more marked horizontal optic disc rotation, and longer disc-fovea distance [26]. In both studies, macular Bruch´s membrane length was not significantly associated with parapapillary gamma zone and vertical disc rotation. The lack of an association between vertical disc rotation and length of macular Bruch´s membrane was in agreement with the findings obtained in another recent investigations, in which the macular Bruch´s membrane length (measured as distance between the fovea and the temporal edge of gamma zone) was not significantly associated with axial length. In contrast, the disc-fovea distance and the width of parapapillary gamma zone strongly increased with longer axial length [18,27]. It also fits with the result that macular retinal thickness was not correlated with axial length [28]. Considering that macular Bruch´s membrane length was independent of axial elongation, the correlations make one infer that the enlargement of the disc-fovea distance and the amount of the vertical optic disc rotation, both occurring in association with axial elongation, were caused by, or associated with, the increase in parapapillary gamma zone (i.e. the Bruch´s membrane free zone).

The finding of our present investigation that the vertical rotation of Bruch´s membrane opening as compared with the vertical rotation of the optic nerve scleral canal opening was significantly (*P*<0.001) more marked suggested that the vertical disc rotation as it appears upon ophthalmoscopy is more due to an arrangement of Bruch´s membrane opening than due to the arrangement of the optic nerve scleral canal. Reason may be that Bruch´s membrane as a solid multilayered double basal membrane as compared to the collagenous tissue of the peripapillary scleral flange is more stiff and resistant to changes. The optic nerve scleral canal defined opening is surrounded by the peripapillary scleral flange which can show a ten-fold elongation and a thinning to less than 10% of its normal values in eyes with high axial myopia as compared to eyes with normal axial length [29]. In contrast to this marked tissue thinning in association with high axial elongation, Bruch´s membrane in markedly axially elongated eyes and in eyes with a normal axial length did not differ in thickness, neither in the equatorial region nor in the macular region [30]. It fits with a notion, that Bruch´s membrane may play a primary role in the biomechanics of axial elongation [31].

Horizontal optic disc rotation (with the superior disc pole coming forward) was associated with an increased disc-fovea angle, indicating an inferior dislocation of the fovea. The reasons for this association have remained unclear so far. Interestingly, horizontal disc rotation was associated with an increased corneal astigmatism in the univariate analysis. It agrees with a former study on an association between corneal astigmatism and the prevalence of so called tilted discs [32]. It suggests that an abnormal shape of the posterior foramen in the eye globe (i.e. the optic disc shape in terms of a horizontal disc rotation) is associated with an abnormal shape of the anterior foramen of the eye globe, i.e. the cornea. Practically, it indicates that in children with a horizontal disc rotation one may perform refractometry to detect an abnormal corneal astigmatism and to prevent amblyopia. The amount of the horizontal disc rotation was not correlated with the parameters of axial length, parapapillary beta/gamma zone width or disc-fovea distance. It fits with the finding that corneal astigmatism is not associated with axial length [33]. Eyes with a horizontally rotated optic disc had a thinner RNFL in the superior nasal region and thicker RNFL in the inferior nasal region. This findings parallels an observation that a larger optic disc-fovea angle correlated with a thinner RNFL in the nasal superior sector, superior sector and temporal superior sector and with a thicker RNFL in the inferior sector, nasal inferior sector and nasal sector [34]. These findings show the importance of taking the optic disc shape (i.e. the horizontal disc rotation) in addition to the disc-fovea angle into account when the profile of the RNFL is examined [34–36].

Based on the findings obtained in our study, future investigations may further explore the potential role the biomechanics of the optic nerve, in particular the biomechanics of the dura mater of the optic nerve, may play for the vertical optic disc rotation and the development and enlargement of parapapillary gamma zone. Recent studies have shown that during horizontal eye movements, the optic nerve pulled the optic nerve head posteriorly [37–39]. It hold true in particular for adductions as compared to abductions. The optic nerve head strains following a lateral eye movement of 13° were large and higher than those resulting from an IOP of 50 mm Hg [37]. Since the optic nerve originates in the nasal upper region of the orbit, the optic nerve pull will be stronger on the temporal side of the optic nerve head than on its nasal side. The optic nerve pull may also be larger at the inferior disc pole than at the superior disc pole. These regional differences could explain the backward movement of the temporal optic disc border leading to the disc rotation around its vertical axis. The regional differences could explain the development of suprachoroidal cavitations preferably at the inferior disc pole region of eyes with a marked vertical optic disc rotation [40–42].

When discussing the results of our study, potential limitations may have to be considered. The definition of the optic disc border to define the angle of optic disc rotation varied between studies. While our reference plane for the Bruch´s membrane opening plane was the line connecting two points at a distance of 1500μm form the optic disc center, Yokoyama and colleagues drew a line passing through the center of the disc from the temporal to the nasal disc edge, Choi and associates used topographic optic disc images, and Hosseini and colleagues connected the inner edges of Bruch membrane on each side of the optic nerve head as the reference plane [7,22,43]. One may also have to take into account that the angle of the optic nerve head image on the OCT screen partially depended on the technique how the image was taken. The probability of having the image inclined to one side versus the other side (temporal versus nasal, and superior versus inferior) was however independent of the side, so that this weakness in the methodology might have increased the noise of the measurements, however not the determination of the mean value. Since despite the noise in the measurements of the amount of the optic disc rotation the associations of disc rotation with other variables were statistically significant, the weakness in the technique may only serve to underline the results and conclusions.

In conclusion, vertical Bruch´s membrane opening rotation was associated with highly myopic axial elongation, increased disc-fovea distance and development or enlargement of parapapillary, Bruch´s membrane free, gamma zone, while macular Bruch´s membrane length was not affected. The vertical rotation of Bruch´s membrane opening was more marked than the vertical rotation of the optic nerve scleral canal defined opening. Horizontal disc rotation was associated with an inferior dislocation of the fovea, in addition to a thinner superior nasal RNFL and thicker inferior nasal RNFL. The latter association may be taken into account in the interpretation of RNFL thickness profiles.
